# High α B-crystallin and p53 co-expression is associated with poor prognosis in ovarian cancer

**DOI:** 10.1042/BSR20182407

**Published:** 2019-06-18

**Authors:** Lin Tan, Ling Sha, Ning Hou, Mei Zhang, Qian Ma, Chuanbing Shi

**Affiliations:** 1 Department of Obstetrics and Gynaecology, Pukou District Central Hospital, Pukou Branch of Jiangsu Province Hospital, The First Affiliated Hospital of Nanjing Medical University, 166 Shanghe street, Nanjing, Jiangsu, China; 2 Department of Neurology, Affiliated Drum Tower Hospital of Nanjing University Medical School, 321 ZhongShan Road, Nanjing, Jiangsu, China; 3 Department of Pathology, Jiangsu Cancer Hospital, Affiliated Cancer Hospital of Nanjing Medical University, 42 Baiziting, Nanjing, Jiangsu, China; 4 Department of Pathology, Pukou District Central Hospital, Pukou Branch of Jiangsu Province Hospital, The First Affiliated Hospital of Nanjing Medical University, 166 Shanghe street, Nanjing, Jiangsu, China; 5 Department of Pathology, The Second Chinese Medicine Hospital of Jiangsu Province, Nanjing, Jiangsu, China

**Keywords:** coexpression, CRYAB, immunohistochemistry, p53, prognosis

## Abstract

Objectives: The present study investigated the correlation between α B-crystallin (CRYAB, HSPB5) and p53 expression in ovarian cancer and further analyzed the relationship between their expression and clinicopathology and the prognostic value of their co-expression in ovarian cancer. Methods: CRYAB and p53 expression was assessed using immunohistochemistry on ovarian cancer tumor tissues from 103 cases and validated in an independent group of 103 ovarian cancer patients. Results: High CRYAB and p53 expression rates in ovarian cancer tissues were 61.17% (63/103) and 57.28% (59/103), respectively, and their expression was positively correlated (r = 0.525, *P*=0.000). High CRYAB expression was significantly correlated with tumor size (*P*=0.028), lymph node metastasis (*P*=0.000), distant metastasis (*P*=0.005), tumor node metastasis (TNM) stage (*P*=0.002), and survival (*P*=0.000), while high p53 expression was significantly correlated with tumor size (*P*=0.006), pathological grade (*P*=0.023), lymph node metastasis (*P*=0.001), and survival (*P*=0.000). Further studies found that the high CRYAB and p53 co-expression was also significantly correlated with pathological grade (*P*=0.024), lymph node metastasis (*P*=0.000), Distant metastasis (*P*=0.015), TNM stage (*P*=0.013), and survival (*P*=0.000). High expression of either CRYAB or p53 and high co-expression of CRYAB and p53 were significantly correlated with poor disease-free survival (DFS) and overall survival (OS), respectively (*P*<0.05). Patients with high CRYAB and p53 co-expression had the worst prognoses among the groups. In addition, multivariate Cox regression models showed that high expression of either CRYAB or p53 and high co-expression of CRYAB and p53 were independent prognostic factors for DFS and OS (*P*<0.05). Moreover, the positive correlation and prognostic value of CRYAB and p53 expression were verified in another independent dataset. Conclusions: We demonstrated that patients with high CRYAB and p53 co-expression in ovarian cancer have significantly increased risks of recurrence, metastasis, and death compared with other patients. Therefore, more frequent follow-up of patients with high CRYAB and p53 co-expression is required. Our results also suggest that combination therapy with CRYAB inhibitors and p53 blockers may benefit future treatment of ovarian cancer patients with high co-expression of CRYAB and p53.

## Introduction

Ovarian cancer is a common malignant gynecological tumor that is difficult to diagnose early, progresses rapidly, and causes high mortality. Each year, 238,700 new ovarian cancer cases are diagnosed worldwide with 151,900 patient deaths [1]. The numbers of new diagnoses and deaths from ovarian cancer in China are 52,100 and 22,500, respectively [2]. Approximately 90% of ovarian cancers are epithelial malignancies in terms of histological type, including serous, mucinous, and endometrioid carcinomas, clear cell adenocarcinoma, and other rare histological types [3]. Among all gynecological tumors, ovarian cancer has the highest mortality rate and the worst prognosis. Approximately 70% of ovarian cancers develop into advanced cancer upon discovery. Although surgical treatment, chemotherapy, radiotherapy, and targeted therapy have developed rapidly in recent years, the 5-year survival for patients with ovarian cancer is only 20% [4,5]. Therefore, new ovarian cancer markers are urgently needed to detect early recurrence and poor prognosis.

The α B-crystallin (CRYAB ), also named heat shock protein B5 (HspB5) was first discovered in the lens and is a member of the small-molecule heat-shock protein family. CRYAB acts as a molecular chaperone, and when cells encounter external stress, such as radiation and peroxidation, CRYAB can bind unfolded proteins, inhibit their aggregation, and prevent degeneration and degradation, thereby promoting cell survival, inhibiting apoptosis, protecting cells and degrading proteases [6]. CRYAB’s role in tumor pathogenesis and development has been studied in recent years. Studies on breast cancer [7,8], head and neck cancer [9], glioma [10], lung cancer [11] and other tumors revealed that CRYAB was associated with tumor prognosis and can be used as an independent indicator of tumor prognosis. CRYAB promotes gastric cancer cell migration and invasion via NF-κB signaling pathway-mediated epithelial–mesenchymal transition [12]. In metaplastic breast cancer, CRYAB was found to be highly expressed and associated with brain metastasis, and silencing the CRYAB gene using RNA interference technology significantly reduced tumor invasion and metastasis ability [7,8]. Our previous study also found that CRYAB was associated with poor prognosis in colorectal cancer and promotes colorectal cancer invasion and metastasis via the epithelial–mesenchymal transition [13,14].

Tumor protein 53 (TP53)’s role as a tumor suppressor gene in tumors has been extensively studied [15,16]. TP53 can initiate the mitochondrial apoptotic pathway by regulating apoptosis-related gene expression, thereby promoting tumor cell apoptosis [17]. TP53 mutation, which is common in cancer cells, indicates poor prognosis in malignant tumors [18] and is of great significance in treating tumors [19]. TP53 mutation has important significance and value in ovarian cancer prognosis and treatment [20,21] and is associated with poor prognosis [22,23]. CRYAB affects p53 mitochondrial translocation during oxidative stress-induced apoptosis [24]. The p53 regulates bidirectional genes and repairs heat shock protein B2 (HspB2)/CRYAB-mediated reactive oxygen species (ROS) and the Warburg effect [25]. CRYAB inhibits cell apoptosis and promotes cell proliferation by binding to p53.

However, the correlation between CRYAB and p53 expression in ovarian cancer remains unknown. Since both CRYAB and p53 are involved in many aspects of tumorigenesis, CRYAB and p53 co-expression may affect the prognoses of patients with ovarian cancer, which may provide a new targeted therapeutic strategy for ovarian cancer. In the present study, we examined CRYAB and p53 expression in tumor tissues from 103 patients with ovarian cancer via immunohistochemistry (IHC) and investigated the relationship between CRYAB and p53 expression and clinicopathological features. The results were verified in another group of 103 ovarian cancer patients.

## Materials and methods

### Patients and specimens

Two hundred and six ovarian cancer specimens were obtained from patients treated at the Jiangsu Cancer Hospital in China between 2004 and 2015. The patients had not been treated before surgery. Histological diagnoses were confirmed postsurgery by pathology per the seventh edition of the American Joint Committee on Cancer’s (AJCC) cancer staging guidelines. Clinical and pathological data were retrospectively obtained from medical records. The patients were divided into two groups, with 103 per group. The research has been carried out in accordance with the World Medical Association Declaration of Helsinki, and that all subjects provided written informed consent. The study was approved by the local medical ethics committee.

### Immunohistochemistry and assessment

CRYAB and p53 protein expression levels in tumors were detected by IHC staining using a two-step method. Formalin-fixed and paraffin-embedded ovarian cancer samples were cut into 4 μm thick sections. After deparaffinization and rinsing, antigen retrieval was accomplished by incubating the sections in pH 6.0 citrate buffer after heating in a microwave oven. Endogenous peroxidase was blocked by incubating the sections in 3% hydrogen peroxide for 5 min, followed by three washes in buffer. Sections were incubated at room temperature for 2 h with primary antibodies against CRYAB and p53 (Abcam, Cambridge, MA, U.S.A.), which were diluted 1:150. The sections were then incubated with secondary antibody (Abcam) for 30 min at room temperature. The negative control was incubated with phosphate-buffered saline. The immunostaining intensity was quantitated using Image J (NIH, Bethesda, MD) software. The following algebraic formula was recommended to calculate the IHC optical density score (from 1 to 4) for the IHC images. IHC optical density score = (Percentage contribution of high positive × 4 + Percentage contribution of positive × 3 + Percentage contribution of low positive × 2 + Percentage contribution of negative × 1)/100 [26].

### Statistical analysis

Statistical analysis was performed using SPSS software (version 23.0; IBM, Armonk, NY, U.S.A.). The χ2 test was used to examine the relationship between CRYAB and p53 expression and the clinicopathological features of ovarian cancer. The Spearman correlation test was used to examine the correlation between CRYAB and p53 expression in ovarian cancer. The relationships between CRYAB and p53 expression levels and disease-free survival (DFS) and overall survival (OS) were determined using Kaplan–Meier analysis. Log-rank tests were used to assess the differences in survival between patient subgroups. The Cox proportional hazard regression model was used to identify potential independent prognostic factors. The *P*<0.05 (two-tailed) was considered statistically significant.

### Independent internal validation cohort

To further validate our results, we examined the correlation and prognostic values of CRYAB and p53 expression in another group of 103 ovarian cancer patients. Experimental methods and statistical analyses were performed in the same manner as in the experimental cohort study.

## Results

### Relationship between CRYAB and p53 expression and clinicopathological features

**Figure 1:**
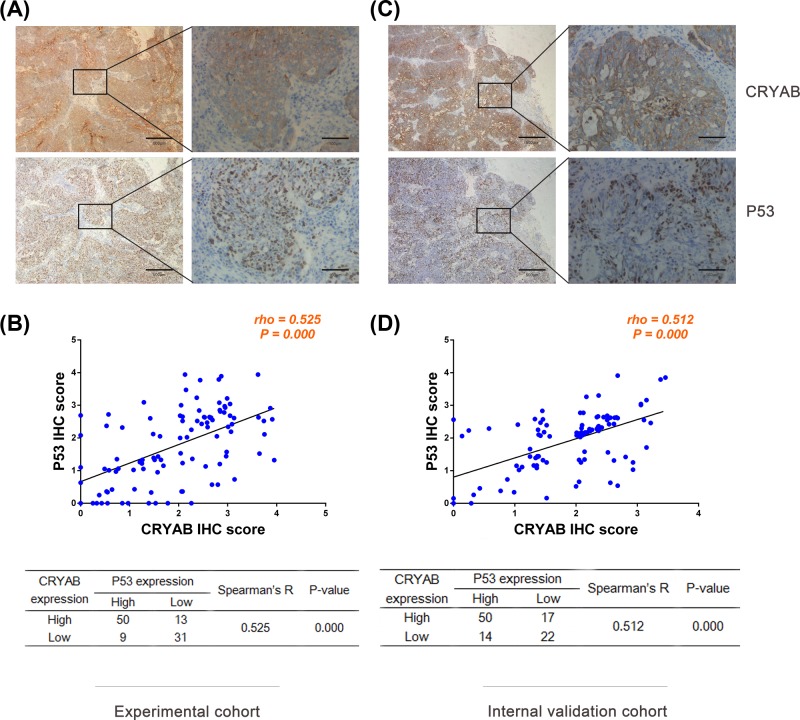
Representative immunohistochemical images of CRYAB and p53 protein expression in ovarian cancer Representative immunohistochemical images of CRYAB and p53 protein expression in ovarian cancer tissues in the experimental cohort (**A**) and validation cohort (**C**). Positive correlations were found between CRYAB and p53 expression in the experimental (**B**) and validation cohorts (**D**). Ovarian cancer patients with higher CRYAB IHC scores had higher p53 IHC scores (**B,D**), and statistical analysis was performed using Spearman correlation analysis as indicated by the scatter plot.

In ovarian cancer tissues, the CRYAB protein was mainly found in the cell membrane and/or cytoplasm, and p53 was mainly expressed in the nucleus (Figure 1A). CRYAB and p53 had high expression rates of 61.17% (63/103) and 57.28% (59/103), respectively, in the ovarian cancer tissues. High CRYAB expression in tumor cells was significantly correlated with tumor size (*P*=0.028), lymph node metastasis (*P*=0.000), distant metastasis (*P*=0.005), tumor node metastasis (TNM) stage (*P*=0.002), and survival (*P*=0.000), while high p53 expression was significantly correlated with tumor size (*P*=0.006), pathological grade (*P*=0.023), lymph node metastasis (*P*=0.001), and survival (*P*=0.000). Further studies found that high CRYAB and p53 co-expression was also significantly correlated with pathological grade (*P*=0.024), lymph node metastasis (*P*=0.000), Distant metastasis (*P*=0.015), TNM stage (*P*=0.013), and survival (*P*=0.000). (Table 1). No statistically significant association was found between CRYAB and p53 expression and other clinicopathological features. Figure 1B shows the relationship between the high CRYAB and p53 expression. Fifty of the 103 patients had high co-expression levels of CRYAB and p53 (48.54%), and 31 of the 103 patients had low or no expression of both proteins (30.10%). In addition, Spearman’s correlation test showed that CRYAB and p53 expression were positively correlated (r = 0.525; *P*=0.000, Figure 1B).

**Table 1:** Relationship between CRYAB and p53 expression status and patient characteristics

Groups	n	CRYAB expression			p53 expression			CRYAB/p53 co-expression		
		High (%)	χ^2^	*P*-value	High (%)	χ^2^	*P-value*	Double High (%)	χ^2^	*P*-value
Total	103	63 (61.17)			59 (57.28)			50 (48.54)		
Age (years)										
≥60	35	22 (62.86)	0.06	0.800	22 (62.86	0.67	0.412	18 (51.43)	0.18	0.674
<60	68	41 (60.29)			37 (54.41)			32 (47.06)		
Tumor size (cm)										
>5	81	54 (66.67)	4.83	0.028*	52 (64.20)	7.41	0.006*	43 (53.09)	3.13	0.077
≤5	22	9 (40.91)			7 (31.82)			7 (31.82)		
Histological type										
1 Serous	67	45 (67.16)	4.09	0.252	43 (64.18)	4.84	0.184	38 (56.72)	5.61	0.132
3 Mucinous	17	9 (52.94)			6 (35.29)			5 (29.41)		
2 Endometroid	15	8 (53.33)			8 (53.33)			6 (40.00)		
4 Clear cell	4	1 (25.00)			2 (50.00)			1 (25.00)		
Pathological grade										
Grade 1	23	15 (65.22)	4.86	0.088	14 (60.87)	7.56	0.023*	13 (56.52)	7.48	0.024*
Grade 2	22	9 (40.91)			7 (31.82)			5 (22.73)		
Grade 3	58	39 (67.24)			38 (65.52)			32 (55.17)		
Lymph node metastasis										
Positive	33	30 (90.91)	18.09	0.000*	27(81.82)	11.95	0.001*	26(78.79)	17.78	0.000*
Negative	70	33 (47.14)			32(45.71)			24(34.29)		
Distant metastasis										
Positive	26	22 (84.62)	8.05	0.005*	19(73.08)	3.55	0.060	18(69.23)	5.96	0.015*
Negative	77	41 (53.25)			40(51.95)			32(41.56)		
TNM stage										
Stage I	2	0 (0.00)	14.94	0.002*	1 (50.00)	4.10	0.251	0 (0.00)	10.79	0.013*
Stage II	20	7 (35.00)			9 (45.00)			5 (25.00)		
Stage III	55	34 (61.82)			30 (54.55)			27 (49.09)		
Stage IV	26	22 (84.62)			19 (73.08)			18 (69.23)		
Survival										
Living	43	12 (27.91)	34.37	0.000*	13 (30.23)	22.07	0.000*	6 (13.95)	35.36	0.000*
Deceased	60	51(85.00)			46 (76.67)			44 (73.33)		

* *P*<0.05.

### Survival analysis

**Figure 2:**
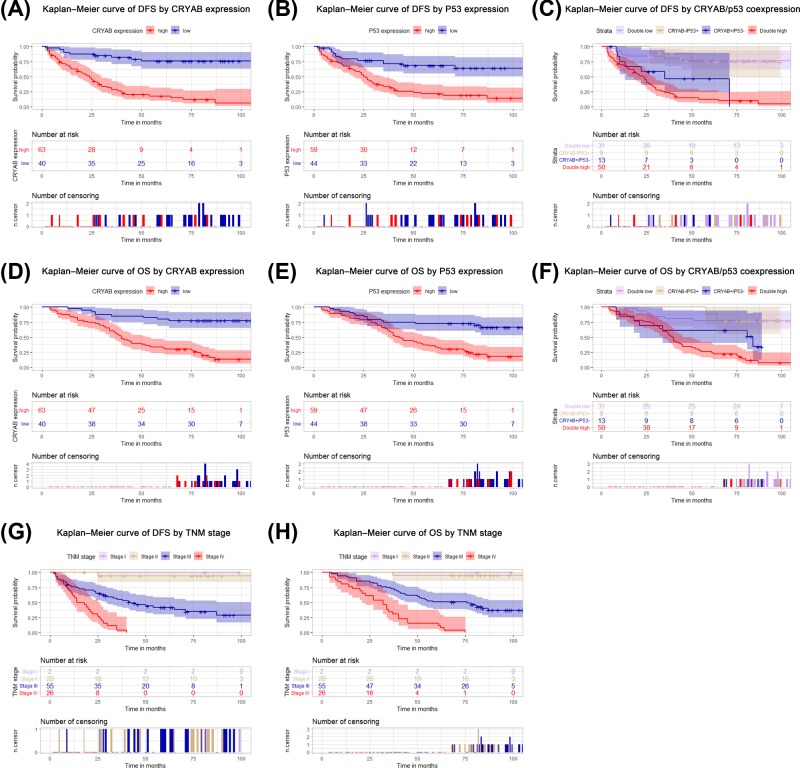
Prognostic value of CRYAB and p53 expression in ovarian cancer tissues Kaplan–Meier analysis was conducted in the experimental group. DFS and OS for CRYAB (**A,B**), p53 (**C,D**), CRYAB and p53 co-expression (**E,F**), and TNM (**G,H**).

We further explored the prognostic value of CRYAB and p53 expression in ovarian cancer, particularly the prognostic value of their co-expression. Kaplan–Meier analysis revealed that patients with high CRYAB expression had shorter DFS (*P*<0.001, Figure 2A) and lower OS (*P*<0.001; Figure 2D) than did patients with low CRYAB expression. In addition, p53 overexpression significantly predicted poor DFS rates (*P*<0.001; Figure 2B) and OS rates (*P*<0.001; Figure 2E). Based on these results, we divided 103 ovarian cancer patients into four subgroups to further conduct Kaplan–Meier analysis to reveal the prognostic value of CRYAB and p53 co-expression. The results showed that DFS and OS differed significantly between subgroups. Stratified Kaplan–Meier analysis further showed that the subgroup with positive CRYAB and p53 co-expression had the worst DFS (*P*<0.001, Figure 2C) and OS (*P*<0.001, Figure 2F) among subgroups. In addition, TNM staging also suggested that patients with TNM stages of Large, high grade cancer had shorter DFS (*P*<0.001; Figure 2G) and OS (*P*<0.001; Figure 2H) than did patients with TNM stages of small, low-grade cancer. Given the significant correlation between CRYAB and p53 expression, we performed multiple univariate and multivariate Cox proportional hazard analyses, including analysis of the groups with high CRYAB expression, high p53 expression, and high expression of both CRYAB and p53. Univariate analysis showed that DFS prognosis was associated with CRYAB expression, histological type, Lymph node metastasis, distant metastasis, and tumor TNM stage. All the above items were also associated with OS in 103 ovarian cancer patients. The multivariate Cox regression model for CRYAB showed that TNM stage (*P*=0.011), and high CRYAB expression (*P*=0.000) were independent indicators of DFS prognosis (Table 2). TNM stage (*P*=0.009), and high CRYAB expression (*P*=0.001) could independently predict adverse OS (Table 2). In addition, multivariate analysis of the p53 expression group confirmed that TNM stage (*P*=0.005), and high p53 expression (*P*=0.001) independently predicted DFS (Table 3). Moreover, TNM stage (*P*=0.004), and high p53 expression (*P*=0.005) independently predicted OS (Table 3). Further, we considered the effects of the combined indicators (CRYAB and p53) on ovarian cancer prognosis. Table 4 shows the results, which suggest that TNM stage and CRYAB and p53 co-expression independently predicted DFS (*P*=0.007; *P*=0.000) and OS (*P*=0.007; *P*=0.001).

**Figure 3:**
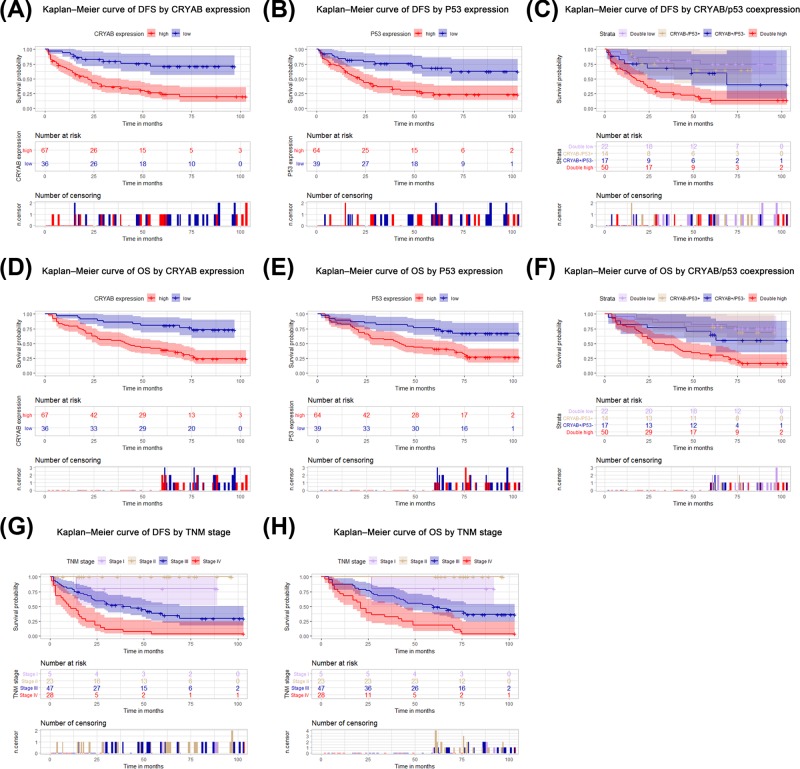
Prognostic value of CRYAB and p53 expression in ovarian cancer tissues Kaplan–Meier analysis was conducted in the validation group. DFS and OS for CRYAB (**A,D**), p53 (**B,E**), CRYAB and p53 co-expression (**C,F**) and TNM (**G,H**).

**Table 2:** Multivariate analysis of CRYAB expression in DFS and OS in patients with ovarian cancer

Variable	Disease-free survival			Overall survival		
	HR	95% CI	*P*-value	HR	95% CI	*P*-value
CRYAB expression (High vs Low)	4.27	1.965–9.280	0.000*	3.63	1.694–7.778	0.001*
Histological type (Serous vs Mucinous vs Endometroid vs Clear cell)	1.23	0.871–1.745	0.238	1.31	0.950–1.811	0.100
Lymph node metastasis (Positive vs Negative)	1.33	0.690–2.567	0.394	1.11	0.555–2.215	0.770
Distant metastasis (Positive vs Negative)	6.48	0.773–54.375	0.085	5.69	0.677–47.800	0.109
TNM stage (Stage I vs Stage II vs Stage III vs Stage IV)	0.07	0.010–0.545	0.011*	0.07	0.010–0.524	0.009*

Abbreviations: 95% CI, 95% confidence interval; HR, heart rate.

* *P*<0.05.

**Table 3:** Multivariate analysis of p53 expression in DFS and OS in patients with ovarian cancer

Variable	Disease-free survival			Overall survival		
	HR	95% CI	*P*-value	HR	95% CI	*P*-value
P53 expression (High vs Low)	2.87	1.498–5.488	0.001*	2.62	1.329–5.170	0.005*
Histological type (Serous vs Mucinous vs Endometroid vs Clear cell)	1.21	0.866–1.695	0.264	1.27	0.928–1.738	0.135
Lymph node metastasis (Positive vs Negative)	1.76	0.863–3.609	0.120	1.33	0.628–2.823	0.454
Distant metastasis (Positive vs Negative)	10.52	1.238–89.434	0.031*	9.06	1.070–76.791	0.043
TNM stage (Stage I vs Stage II vs Stage III vs Stage IV)	0.06	0.008–0.409	0.005*	0.05	0.007–0.398	0.004*

Abbreviations: 95% CI, 95% confidence interval; HR, heart rate.

* *P*<0.05.

**Table 4:** Multivariate analysis of CRYAB and p53 co-expression in DFS and OS in patients with ovarian cancer

Variable	Disease-free survival			Overall survival		
	HR	95% CI	*P*-value	HR	95% CI	*P*-value
Expression of CRYAB and p53 (Double-positive vs Others)	3.46	1.830–6.528	0.000*	3.12	1.634–5.965	0.001*
Histological type (Serous vs Mucinous vs Endometroid vs Clear cell)	1.17	0.832–1.642	0.370	1.25	0.916–1.714	0.157
Lymph node metastasis (Positive vs Negative)	1.64	0.811–3.315	0.168	1.24	0.591–2.604	0.570
Distant metastasis (Positive vs Negative)	8.98	1.064–75.762	0.044	7.25	0.862–60.975	0.068
TNM stage (Stage I vs Stage II vs Stage III vs Stage IV)	0.06	0.009–0.473	0.007*	0.07	0.009–0.477	0.007*

Abbreviations: 95% CI, 95% confidence interval; HR, heart rate.

* *P*<0.05.

### Independent internal validation cohort

In the internal validation group, the CRYAB and p53 distributions in tumor cells were the same as those in the experimental group. Representative CRYAB and p53 immunohistochemical staining images are shown in Figure 1C. The high CRYAB and p53 expression rates observed in ovarian cancer were 65.05% (67/103) and 62.14% (64/103), respectively. CRYAB expression was significantly elevated in ovarian cancer patients with histological type with serous carcinomas (*P*=0.018), positive lymph node metastasis (*P*=0.001), positive distant metastasis (*P*=0.002), TNM stages of large, high grade cancer (*P*=0.000), and lower survival (*P*=0.000). The p53 expression was significantly elevated in ovarian cancer patients with positive lymph node metastasis (*P*=0.003), positive distant metastasis (*P*=0.036), TNM stages of large, high grade cancer (*P*=0.003) and lower survival (*P*=0.000). In the internal validation cohort (Supplementary Table S1), high CRYAB and p53 co-expression was significantly associated with histological type with serous carcinomas (*P*=0.001), positive lymph node metastasis (*P*=0.000), positive distant metastasis (*P*=0.005), TNM stages of large, high grade cancer (*P*=0.000), and lower survival (*P*=0.000) but was not statistically significantly associated with other clinicopathological features; (Supplementary Tables S1). Spearman correlation analysis showed that CRYAB and p53 expression was significantly correlated (*r* = 0.512; *P*=0.000) (Figure 1D). The prognostic values of high CRYAB or p53 expression and high CRYAB and p53 co-expression were verified via Kaplan–Meier survival analysis of DFS and OS in another 103 patients with ovarian cancer (*P*<0.05; Figure 3A-H). In addition, Cox proportional risk analysis showed that high CRYAB and p53 co-expression (*P*=0.005) independently predicted DFS. However, high CRYAB expression (*P*=0.062) and high p53 expression (*P*=0.069) in DFS was not statistically significant in the Cox proportional hazard analysis (Supplementary Tables S2–S4). In the multivariate model, high CRYAB expression, high CRYAB and p53 co-expression and TNM staging independently predicted OS (*P*<0.01; Supplementary Tables S2–S4). However, high p53 expression in OS was not statistically significant in the Cox proportional hazard analysis (*P*=0.106; Supplementary Table S3).

## Discussion

CRYAB is the B subunit of α-crystallin with a molecular weight of 20 kDa. It is an important member of the crystallin family of proteins and belongs to the small-molecule heat-shock protein family. CRYAB is involved in cell development, differentiation and proliferation and inhibits apoptosis [27]. CRYAB is associated with poor prognosis in various tumors and is a potential target for their treatment [28]. High CRYAB expression represents an independent molecular marker for unfavorable outcomes in ovarian cancer patients and impairs TRAIL- and cisplatin-induced apoptosis in human ovarian cancer cells [29]. The p53 overexpression is the main carcinogenic factor in ovarian cancer [23]. The p53, cyclin D1, and p21-Waf1/Cip1 expression predict poor clinical outcomes in serous epithelial ovarian cancer [22]. CRYAB binds the p53 tumor suppressor gene product, preventing its translocation to the mitochondria [24], which would likely inhibit the gene’s ability to induce Bax-dependent mitochondrial outer membrane permeabilization. In addition, CRYAB has been reported to promote p53 degradation by forming an Fbx4-αB-crystallin E3 ubiquitin ligase that marks p53 for proteasomal degradation [30]. CRYAB binds with p53 to inhibit mitochondrial outer membrane permeabilization and subsequent caspase activation, thereby inhibiting apoptosis and promoting cell proliferation.

In the present study, we demonstrated that ovarian cancer patients with high CRYAB or p53 expression have significantly high risks of recurrence, metastasis and death, which is consistent with previous studies [23,28,29]. We, for the first time, discovered a positive correlation between CRYAB and p53 expression in ovarian cancer tumor tissues. High CRYAB and p53 co-expression was significantly correlated with pathological grade, lymph node metastasis, distant metastasis TNM stage, and survival. Moreover, ovarian cancer patients with high CRYAB and p53 co-expression had the worst prognoses. Multivariate survival analysis further supported that high CRYAB and p53 co-expression in ovarian cancer tissues was an independent prognostic factor for DFS and OS. This prognostic value was further validated in an independent cohort of another group of 103 ovarian cancer patients.

Although our study found that CRYAB and p53 are positively correlated in ovarian cancer, whether CRYAB selectively acts on the TP53 gene in ovarian cancer, promotes p53 overexpression, and thus promotes ovarian cancer invasion and metastasis requires further study.

In summary, we, for the first time, report that CRYAB and p53 expression is positively correlated in ovarian cancer, high CRYAB and p53 co-expression is an independent prognostic factor of DFS and OS, and patients with high CRYAB and p53 co-expression have the worst prognoses among ovarian cancer patients. Therefore, patients with high CRYAB and p53 co-expression require more frequent follow-up. Combination therapy that applies both CRYAB inhibitors and p53 blockers may benefit ovarian cancer patients with high CRYAB and p53 co-expression. Related research work is in progress.

## Supporting information

**Supplemental Table S1:** Relationship between CRYAB and p53 expression status and patient characteristics in the validation cohort.

**Supplemental Table S2:** Multivariate analysis of CRYAB expression in DFS and OS in patients with ovarian cancer in the validation cohort.

**Supplemental Table S3:** Multivariate analysis of p53 expression in DFS and OS in patients with ovarian cancer in the validation cohort.

**Supplemental Table S4:** Multivariate analysis of CRYAB and p53 coexpression in DFS and OS in patients with ovarian cancer in the validation cohort.

## Abbreviations

95% CI: 95% confidence interval
CRYAB: α B-crystallin
DFS: disease-free survival
HR: hazard ratio
HspB2: heat shock protein B2
HspB5: heat shock protein B5
IHC: immunohistochemistry
IHS: immunohistochemistry score
OS: overall survival
ROS: reactive oxygen species
TNM: tumor node metastasis
TP53: Tumor protein 53
