# Supplementary material for: High α B-crystallin and p53 co-expression is associated with poor prognosis in ovarian cancer
Source: Biosci Rep. 2019 Jun 18;39(6):BSR20182407. doi: 10.1042/BSR20182407 (PMC6579977; doi:10.1042/BSR20182407)
Supplement: Supplementary file 1 [file bsr20182407_Supp1.pdf]

Table 1. Relationship between CRYAB and p53 expression status and patient characteristics in the validation cohort.

Groups	n	CRYAB expression			p53 expression			CRYAB/p53 coexpression		
		High(%)	χ2	p	High(%)	χ2	p	Double High(%)	χ2	p
Total	103	67(65.05)			64(62.14)			50(48.54)		
Age (years)										
≥60	33	24(72.73)	1.26	0.262	24(72.73)	2.32	0.128	19(57.58)	1.59	0.208
<60	70	43(61.43)			40(57.14)			31(44.29)		
Tumor size (cm)										
>5	76	51(67.11)	0.54	0.463	50(65.79)	1.64	0.200	38(50.00)	0.25	0.620
≤5	27	16(59.26)			14(51.85)			12(44.44)		
Histological type										

Serous	58	44(75.86)	10.03	0.018*	42(72.41)	6.93	0.074	37(63.79)	16.32	0.001*
Mucinous	18	8(44.44)			8(44.44)			4(22.22)		
Endometroid	21	10(47.62)			10(38.10)			5(23.81)		
Clear cell	6	5(83.33)			4(66.67)			4(66.67)		
Pathological grade										
Grade 1	19	13(68.42)	1.73	0.420	12(63.16)	0.01	0.995	10(52.63)	0.29	0.863
Grade 2	29	16(55.17)			18(62.07)			13(44.83)		
Grade 3	55	38(69.09)			34(61.82)			27(49.09)		
Lymph node metastasis										
Positive	37	32(86.49)	11.67	0.001*	30(81.08)	8.81	0.003*	28(75.68)	17.02	0.000*
Negative	66	35(53.03)			34(51.52)			22(33.33)		
Distant metastasis										

Positive	28	25(89.29)	9.94	0.002*	22(78.57)	4.42	0.036*	20(71.43)	8.06	0.005*
Negative	75	42(56.00)			42(56.00)			30(40.00)		
TNM stage										
Stage I	5	4(80.00)	23.27	0.000*	3(60.00)	13.76	0.003*	3(60.00)	21.16	0.000*
Stage II	23	6(26.09)			7(30.43)			2(8.70)		
Stage III	47	32(68.09)			32(68.09)			25(53.19)		
Stage IV	28	25(89.29)			22(78.57)			20(71.43)		
Survival										
Living	46	19(41.30)	20.61	0.000*	19(41.30)	15.33	0.000*	9(19.57)	27.94	0.000*
Deceased	57	48(84.21)			45(78.95)			41(71.93)		

*$P < 0.05$

Table 2. Multivariate analysis of CRYAB expression in DFS and OS in patients with ovarian cancer in the validation cohort.

Variable	Disease-free survival			Overall survival		
	HR	95% CI	*P*	HR	95% CI	*P*
Cryab expression (High vs. Low)	2.07	0.964-4.426	0.062	2.54	1.202-5.351	0.015*
Histological type (Serous vs. Mucinous vs. Endometroid vs. Clear cell)	1.03	0.742-1.427	0.862	1.09	0.814-1.465	0.556
Pathological grade (Grade 1 vs. 2 vs. Grade 3)	0.94	0.644-1.381	0.764	0.90	0.597-1.343	0.593
Lymph node metastasis (Positive vs. Negative)	1.11	0.570-2.164	0.759	1.05	0.532-2.071	0.889
Distant metastasis (Positive vs. Negative)	2.59	0.528-12.718	0.241	3.05	0.620-15.012	0.170
TNM stage (Stage I vs. Stage II vs. Stage III vs. Stage IV)	0.16	0.038-0.641	0.010*	0.144	0.035-0.599	0.008*

*$P < 0.05$

Table 3. Multivariate analysis of p53 expression in DFS and OS in patients with ovarian cancer in the validation cohort.

Variable	Disease-free survival			Overall survival		
	HR	95% CI	*P*	HR	95% CI	*P*
P53 expression (High vs. Low)	1.86	0.953-3.646	0.069	1.73	0.889-3.379	0.106
Histological type (Serous vs. Mucinous vs. Endometroid vs. Clear cell)	1.02	0.737-1.418	0.895	1.07	0.798-1.439	0.645
Pathological grade (Grade 1 vs. 2 vs. Grade 3)	0.91	0.620-1.343	0.643	0.91	0.600-1.369	0.640
Lymph node metastasis (Positive vs. Negative)	1.23	0.612-2.454	0.565	1.14	0.557-2.330	0.720
Distant metastasis (Positive vs. Negative)	2.68	0.536-13.365	0.230	3.40	0.676-17.068	0.138
TNM stage (Stage I vs. Stage II vs. Stage III vs. Stage IV)	0.15	0.035-0.604	0.008*	0.13	0.030-0.539	0.005*

* $P < 0.05$

Table 4. Multivariate analysis of CRYAB and p53 coexpression in DFS and OS in patients with ovarian cancer in the validation cohort.

Variable	Disease-free survival			Overall survival		
	HR	95% CI	*P*	HR	95% CI	*P*
Expression of CRYAB and p53 (Double-positive vs. Others)	2.50	1.324-4.733	0.005*	2.63	1.392-4.951	0.003*
Histological type (Serous vs. Mucinous vs. Endometroid vs. Clear cell)	1.05	0.759-1.447	0.776	1.05	0.788-1.403	0.734
Pathological grade (Grade 1 vs. 2 vs. Grade 3)	0.85	0.574-1.252	0.406	0.80	0.528-1.219	0.302
Lymph node metastasis (Positive vs. Negative)	1.06	0.537-2.083	0.872	1.05	0.522-2.116	0.890
Distant metastasis (Positive vs. Negative)	2.44	0.499-11.958	0.270	2.76	0.560-13.602	0.212
TNM stage (Stage I vs. Stage II vs. Stage III vs. Stage IV)	0.16	0.040-0.671	0.012*	0.15	0.037-0.640	0.010*

*$P < 0.05$
